# Large language models improve annotation of viral proteins

**DOI:** 10.21203/rs.3.rs-2852098/v1

**Published:** 2023-05-02

**Authors:** Zachary N. Flamholz, Steve J. Biller, Libusha Kelly

**Affiliations:** 1 Department of Systems and Computational Biology, Albert Einstein College of Medicine; Bronx, NY, USA; 2 Department of Biological Sciences, Wellesley College; Wellesley, MA USA; 3 Department of Microbiology and Immunology, Albert Einstein College of Medicine; Bronx, NY, USA

## Abstract

Viral sequences are poorly annotated in environmental samples, a major roadblock to understanding how viruses influence microbial community structure. Current annotation approaches rely on alignment-based sequence ho-mology methods, which are limited by available viral sequences and sequence divergence in viral proteins. Here, we show that protein language model representations capture viral protein function beyond the limits of remote sequence homology by targeting two axes of viral sequence annotation: systematic labeling of protein families and function identification for biologic discovery. Protein language model representations capture protein functional properties specific to viruses and expand the annotated fraction of ocean virome viral protein sequences by 37%. Among unannotated viral protein families, we identify a novel DNA editing protein family that defines a new mobile element in marine picocyanobacteria. Protein language models thus significantly enhance remote homology detection of viral proteins and can be utilized to enable new biological discovery across diverse functional categories.

Viruses of microbes, hereafter, ‘viruses’, are abundant in the environment and have wide-ranging impacts on microbial communities. Most of what we know about viral diversity, ecology, and function comes from analysis of sequences obtained from environmental samples, yet viruses are difficult to identify, classify, and annotate. Thus, we make statements about viral impacts on microbial community structure and function based on a tiny fraction of viral sequences with sufficient similarity to existing references. In recent years, next-generation sequencing and increasing computational resources have been applied to catalogue the world’s virome^1–7^. While there has been substantial methodological progress in identifying viral DNA in whole community metagenomic sequence data^8–16^, sequence feature annotation and overall taxonomic assignment of identified uncultivated viral genomes (**UViGs**) has lagged considerably. Viruses have no conserved marker genes to enable broad, unified, taxonomic analysis and thus most of the hundreds of thousands of new viruses uncovered in viral catalogue studies remain unclassified^1–7^. Viral taxonomic classification is generally based on using predicted UViG proteins as features for clustering-based^17–19^ or machine learning-based^20^ taxonomic classification. Yet, as many as 86% of environmental viral protein clusters match uncharacterized protein families or have no hits at all^6,7,16,21,22^. Improved annotation of viral protein families (**VPFs**) is thus a necessary, unrealized, step towards understanding the roles of viruses in microbial ecology.

Viral protein annotation currently relies on sequence homology using state-of-the-art profile Hidden Markov Model (**pHMM**)-based approaches. For viral metagenomics, sequence homology methods suffer from two fundamental limitations: (1) the limited library of annotated viral protein sequences from which to construct probabilistic sequence models and (2) the rate at which viral proteins change, quickly diverging beyond recognition by traditional sequence homology metrics. An alignment-free method that does not depend on constructing sequence profiles for statistical sequence homology and that can leverage functional homology between proteins could overcome both challenges.

Advances in the field of natural language processing have increasingly been utilized to identify viral sequences in whole community sequencing data, including k-mer frequency^9,11^ and learned vector representation^10,16,23,24^ methods. In natural language processing, current state-of-the-art large language models are trained in an unsupervised manner on gigantic corpora of text. Recently, this approach has been used to train protein language models (**PLMs**) on billions of protein sequences to learn real number vector representations of amino acids. PLMs capture physico-chemical properties of amino acids and can resolve protein structural and functional information from sequence input alone^25–30^. Unlike sequence, structure and function of viral proteins are better maintained over evolutionary time due to biochemical and fitness constraints^31,32^. We hypothesized that annotating VPFs based on functional homology captured in PLM-based protein representations, rather than strict protein sequence homology, would improve VPF annotation. Therefore, we developed a PLM-based viral protein function classifier and asked if it could improve the viral protein annotation problem.

Using curated VPF databases and recently published PLMs, we show that PLM-based representations of viral protein sequences can capture viral functional homology beyond remote sequence homology. Our analysis focuses on the two axes of viral sequence annotation: systematic labeling of protein families and specific function identification for biologic discovery. The first is the ability to characterize VPFs in environmental viral profiling studies, where we utilize our PLM-based viral classifier to expand the annotated fraction of VPFs collected from the ocean virome by 37%. The second is the functional characterization of specific proteins in UViGs of biological interest, which we demonstrate by using our functional classifier to identify novel phage-like DNA editing proteins. Finally, we show that the PLM-based representations capture functional groupings unique to viruses. PLMs thus capture features of viral proteins that aid in detecting remote homology, a necessary step toward understanding the functions of viral populations across the world.

## Results

### Protein language models capture viral protein function

To determine whether PLMs can aid in annotating viral proteins, it is necessary to determine that PLMs capture properties of viral protein function and that they can identify sequence homology that is invisible to state-of-theart approaches to identify distantly related sequences such as pHMMs. We based our efforts on the Prokaryotic virus Remote Homologous Groups (PHROGs) database, a curated library of VPFs constructed to capture remote sequence homology and manually annotated to high-level functional categories^21^. Because the database was constructed to maximize remote sequence homology captured by each family, it is an ideal dataset to determine whether PLM-based representations can capture viral protein function beyond sequence homology. PHROGs contains 868,340 protein sequences clustered to 38,880 families, of which 5,088 are annotated to 9 functional classes (Supplemental Table 1).

**Figure 1:**
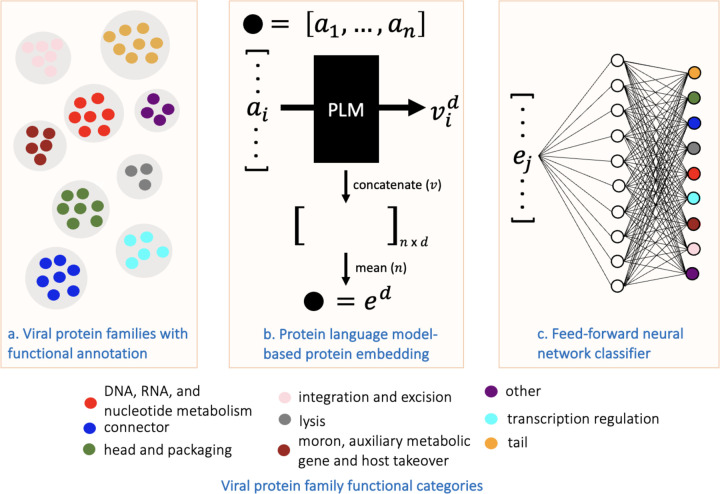
Overview of training viral protein family (VPF) function classifier using protein language models (PLMs). (a) VPFs collected from the PHROGs database with manual annotation to 9 functional categories^21^. (b) Protein sequences are embedded using trained PLMs by averaging amino acid (*a*) vectors (*v*) to a single embedding vector (*e*) of dimension *d*, and (c) used as input to a feed-forward multi-class classifier.

**Figure 2:**
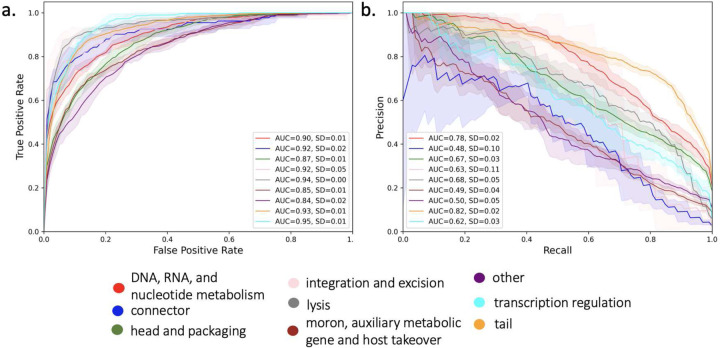
Functional category classification of PHROGs VPFs with PLM-based protein embeddings. (a) Receiver operating characteristic curve with average area under curve (AUC) and standard deviation (SD) over five folds. (b) Precision-recall curve with AUC and SD over five folds. Per fold, training is performed over all proteins in a family and testing is performed on a random single sequence from test families. Protein sequences were embedded using the Transformer_BFD PLM and the classifier consists of a three hidden layer dense neural network and an output layer with softmax activation.

We built our classification model by embedding proteins in annotated families in the PHROGs database to a distributed representation using a PLM. Using a feed-forward neural network with sequence embeddings as input (Figure 1), a functional annotation classifier was trained on VPFs to predict the functional category of sequences from held out VPFs. Five-fold cross validation over the entire annotated set was performed with proteins embedded using four trained PLMs. The PLM trained exclusively with the unsupervised objective of predicting masked amino acids on over 2.1 billion protein sequences from the Big Fantastic Database (**BFD**) (Transformer BFD^28^) performed the best of the PLMs evaluated^28–30^ (Supplemental Table 2, Supplemental Figure 1) and is used for subsequent experiments. Performance with Transformer_BFD for each category is shown in Figure 2. The multiclass classifier achieved an average AUROC of 0.90 and average AUPRC of 0.62 across all classes and folds.

A single classification model was then trained on all annotated families as well as 14,280 families of the unknown function category in order to capture sequences that do not match the functional categories. Subsequent to classifier training, 57 PHROGs families were reclassified. The classifier correctly predicted the re-annotation of 38/57 families (66.6%) despite being trained on the previous incorrect annotation for those families (Supplemental Table 3). The performance on the reannotated families serves as a validation of the classifier’s ability to capture function. Our trained classifier is available for download (https://github.com/kellylab/viral_protein_function_plm).

### Language model protein embeddings capture phage biology

**Figure 3:**
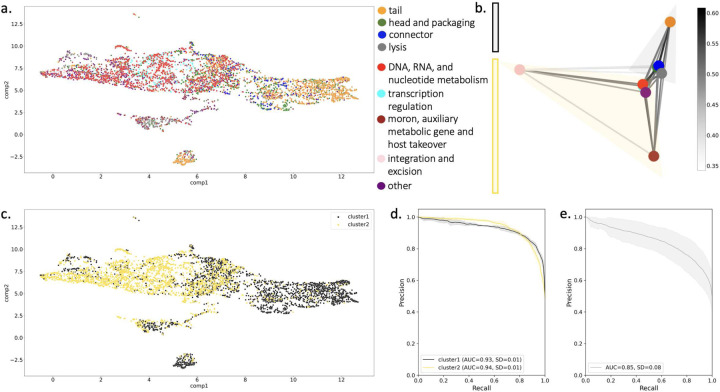
Investigation of PLM-based embedding of PHROGs VPFs. (a) umap projection of PHROG VPFs. VPFs are represented as the centroid of sequence vectors. (b) Spectral network visualization of the inter-category family-family similarity (edge weight), which is measured as the mean family-family centroid similarity across all family pairs between two categories. The category-category similarity matrix is clustered with n=2 into two groups (black and yellow). (c) Spectral clusters are used to color PHROGs VPF umap projection. (d) Clusters are used as binary classes for PHROGs VPF classifier as in 2B. (e) Classifier performance on 10 random two group splits with AUPRC averaged over groups and splits.

Having determined that PLM-based representations of viral proteins can predict function, the viral protein embedding space was investigated to understand what enables the PLM to detect differences between functions. Because a PLM can produce a dense vector representation for any protein sequence, we can interrogate the similarity of sequences in a family and families in a functional category using vector similarity. VPFs were represented as the centroid of sequence embeddings for constituent proteins and visualized for the functionally annotated PHROGs subset (Figure 3a).

While the sequence-sequence vector similarity in families across all categories is high (Supplemental Figure 2a), the intra-category family-family similarity varies between functional categories (Supplemental Figure 2b). Families in the transcription regulation category are most similar, with a median family-family similarity of 0.68, while the integration and excision category had lowest median family-family similarity of 0.51. To ask if there are groupings of categories in the embedding space, we first measured the category-category similarity as the average of the family-family vector similarity for all pairs of families between the categories (Supplemental Figure 3). We spectrally clustered the category-category distance matrix (Figure 3b), revealing a biologically meaningful partition of functional categories into those relating to phage virion structure and infection (cluster1) and those relating to viral genome replication and other host derived genes (cluster2). The partition is apparent when visualizing the PHROGs embedding space (Figure 3c). Grouping of the functional categories into the two clusters identified greatly increases the performance of the PHROGs classifier in five-fold cross validation (Figure 3d) when compared to individual classes (Figure 2B) and when compared to random partitions of the categories into groups of two (Figure 3e).

The ability to classify phage structural proteins, termed phage virion proteins (**PVPs**), is important for identifying and grouping novel sequences, and a number of methods have been recently developed to tackle this problem^33,34^. With the high performance of our cluster1 vs. cluster2 binary classifier, we evaluated how PLM-based classification compares with existing methods for PVP classification. Using a PVP identification task designed previously^34,35^, our method achieved on par performance with state-of-the-art approaches (Table 1).

### Improved classification of proteins from the global ocean virome

**Figure 4:**
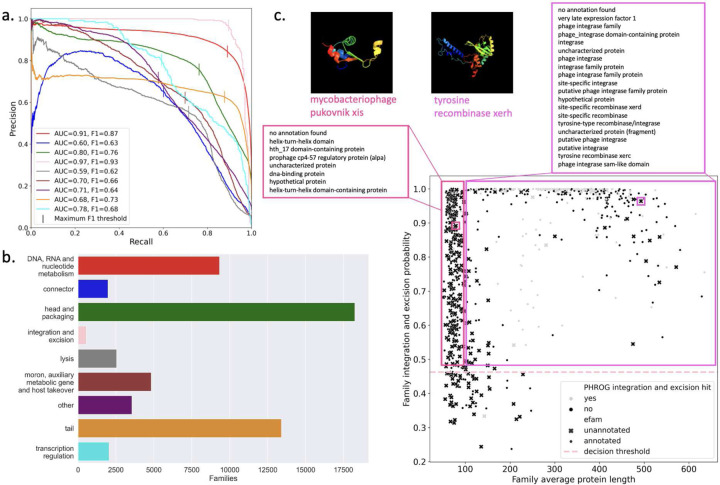
Functional category classifier validation and discovery with the EFAM database of VPFs curated from the ocean virome. (a) Precision-recall curve for EFAM VPFs labeled with PHROGs HMMs and predicted with the PLM-based functional classifier. Performance is measured with AUPRC and optimal F1-score. (b) Number of VPFs in EFAM that are labeled to each functional category based on the category-specific optimal threshold and not captured by PHROGs HMMs. (c) EFAM VPFs predicted integration and excision probability as a function of average protein length in the VPF. Annotation of excisionase (pink) and integase/recombinase (purple) terms are for VPFs annotated in EFAM (·). Structural prediction for two EFAM VPFs that do not match PHROGs HMMs and are unannotated in EFAM (x) are shown with predicted structure, one excisionase (cluster122519) and one integrase (cluster86903). Decision probability is the threshold of maximal F1 for integration and excision category prediction.

To further validate the trained functional classifier, we evaluated the performance of the classifier against pHMM annotation of the largest pan-ecosystem VPF database, EFAM, curated from UVIGs identified in the global oceans^22^ (Supplemental Data 1). With different phage genome sources for database construction, viral genomes in EFAM are not present in the PHROGs training sequences, making this dataset well-suited for an external validation of our classifier. To assign true functional categories to the EFAM VPFs, we used profile-profile HMM matching with the PHROGs database (Supplemental Data 2). 80,942/240,311 (33.7%) of EFAM VPFs were assigned a PHROGs function and these VPFs were predicted using our PLM-based functional classifier trained on PHROG VPFs (Figure 4a). The weighted average F1 score across all categories was 0.70 and is increased to 0.75 when the unknown function category is excluded (Supplemental Table 4).

To highlight the systematic annotation capability of our approach, the optimal F1 decision probability per category was used to predict the functional category of EFAM VPFs not captured by the PHROGs HMMs (Figure 4b). In total we expand the annotated fraction of EFAM by 39,258 families, a 37.8% increase over the number annotated internal to the EFAM database supplemented with annotation by PHROGs (103,919 families).

### PLMs enable identification of novel phage DNA editing enzymes

Integration and excision had the best prediction performance, therefore, VPFs in EFAM labeled to this category were used to highlight the ability of PLM-representations to uncover novel biology. Additionally, detection of genes associated with phage integration into host genomes is crucial in viral bioinformatics for the characterization of phage genomes as temperate. EFAM VPFs predicted in this category can be stratified based on their annotation in the EFAM database itself, with VPFs having average protein lengths >100 matching annotation to known integrase/recombinase proteins and VPFs with average protein lengths <100 matching known excisionases (Figure 4c). We validated our integration and excision prediction for EFAM VPFs that were not annotated in EFAM or by PHROGs HMM matching using structure and domain predictions (Supplemental Table 5). Further investigation of predicted EFAM integrase families led to the annotation of an integrase (cluster86903) on a previously reported putative prophage in uncultured Alphaproteobacteria^36^ highlighting the utility of this approach.

**Figure 5:**
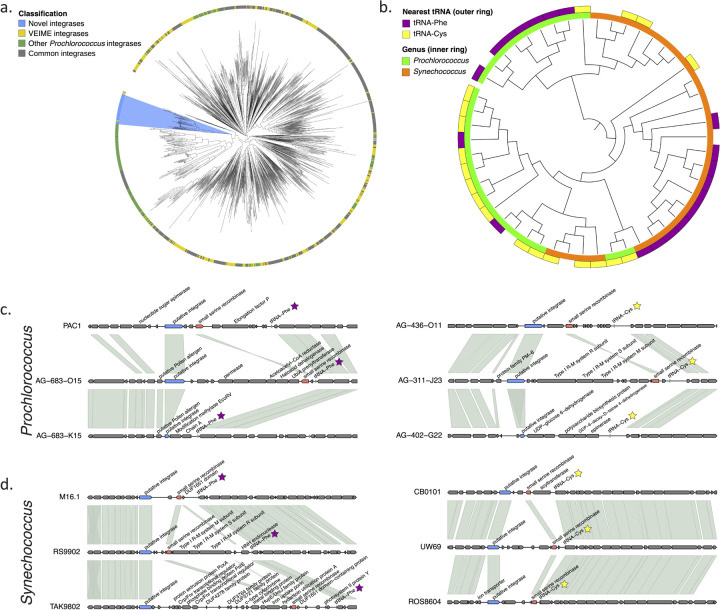
Identification of a novel integrase/recombinase within marine picocyanobacteria. (a) Phylogenetic relationship of the novel integrases (blue) in comparison with tyrosine recombinases described in marine viral parasites (VEIMES; yellow), cyanobacterial mobile element integrases (green) and classes commonly found among well-described phage and mobile elements (e.g. IS, PICIs, ICEs). (b) Phylogenetic groupings of full-length (>350aa) novel integrases in *Prochlorococcus* and *Synechococcus*, in relationship to the closest downstream tRNA (outer ring) and genome taxonomy (inner ring). Gaps reflect unknown tRNA associations from limitations of genome assemblies. (c and d) Genomic context of the novel integrase in selected marine *Prochlorococcus* and *Synechococcus* genomes, respectively. Colored genes indicate the novel integrase (blue), a small serine recombinase frequently found near the integrase (red) and the downstream tRNA (purple or yellow). Shaded regions connect orthologous genes.

Our method was also able to annotate related genes in non-viral contexts. We identified a novel integrase family (cluster158946) located within marine picocyanobacterial genomes, including members of the globally abundant cyanobacteria *Prochlorococcus* and *Synechococcus*. Phylogenetic analysis revealed these enzymes as a novel subgroup within the tyrosine integrase/recombinase family of site-specific integrases. These cyanobacterial integrases are distinct from other recombinases commonly seen in phages and bacterial mobile genetic elements, integrases recently described as being associated with Tycheposon mobile elements in *Prochlorococcus*^37^, or tyrosine recombinases associated with VEIME phage satellites^38^ (Figure 5a). This protein has a different domain structure than is typical of many tyrosine integrases^39^, yet structural modeling confirmed that this enzyme retains the key catalytic residues required for activity^40^ (Supplemental Figure 4). While found only within a subset of available *Prochlorococcus* and *Synechococcus* genomes, where identified the integrase is typically found upstream of one of two specific tRNAs, either tRNA-Phe or tRNA-Cys. tRNAs are frequent integration sites for mobile genetic elements^41^ and phylogenetic groupings of these enzymes correlate with their respective tRNA (Figure 5b), suggesting that these may represent the integration site. The integrases are located in islands of variable genetic content and are also frequently, though not exclusively, found near a small serine recombinase (Figure 5c–d). Together, these properties suggest that this novel enzyme defines a mobile genetic element within marine picocyanobacteria.

## Discussion

Improving viral protein annotation in environmental samples is a vital step towards understanding how viruses influence microbial community structure. Current approaches annotate on average less than 30% of viral protein families in large, global, environmental metagenomes^6,7,16,21,22^, meaning that our understanding of viruses of microbes is based on a small fraction of phage genomes. Annotating viral proteins is also key to studies of viral evolution^42^, characterization of isolate genomes^43^, and to understand the role of viruses as disseminators of DNA in microbial populations^44^.

Our work provides a proof of concept that high-level viral function can be learned with PLM-based representations and extends remote homology detection beyond the ability of universally used, state-of-the-art, alignment-based methods. Furthermore, we used our PLM classifier to discover novel biology in the oceans by interrogating predictions in the integration and excision category, unveiling a previously unrecognized DNA-editing enzyme in the globally abundant marine phototrophs *Proclorococcus* and *Synechococcus* that anchors a putative novel mobile genetic element.

We show that across all nine categories in PHROGs, a single multi-class classifier was able to learn viral function in a stratified five-fold cross validation across the annotated PHROGs VPFs. How does performance differ by category? ”Tail” and ”DNA, RNA, and nucleotide metabolism” were the best performing categories and had the highest number of families, though not the highest number of sequences per family. Two of the worst performing categories, ”moron, auxiliary metabolic gene and host takeover” and ”other”, are the categories that are least specific in their annotation, grouping sequence diverse and functionally distinct families into single categories. These categories also have the lowest number of average sequences per family, which may contribute to their worse performance. While the ”integration and excision” class had the least number of families and the least number of sequences, it had performance similar to ”head and packaging” and ”transcription regulation”. It remains an open question how family sequence diversity and number of sequence contribute to classifier performance. However, that our multi-class classifier can predict functional labels of held out VPFs demonstrates our classifier has captured underlying homology beyond what is captured with alignment-based approaches.

PLM training on the BFD^45^, the largest existing protein corpus that contains protein sequences from uncultivated genomes from metagenomic sequencing data, resulted in the best viral protein function classifier performance. Interestingly, supplemental supervision tasks in PLM training related to structure, such as predicting residue contacts in protein structures^30^, or function, such as protein GO term annotation^29^, did not result in better classification performance. It is possible that this is due to the dearth of viral protein representation in protein structure and knowledge databases. Future work is necessary to determine if there are viral-specific supervised tasks that can enhance PLM training.

A major advantage to an alignment-free classification is the ability to make predictions even when a VPF does not share sequence homology with known proteins/families. To ask if our classifier could improve VPF annotation, we used VPFs from the ocean virome curated in the EFAM database. Using PHROGs HMM-labeled EFAM VPFs as ground truth, our classifier achieved a weighted F1 score of 0.75 for functional annotation.

To ask if our classifier could uncover novel biology, we interrogated newly annotated EFAM VPFs of the integration and excision category, which had the highest predictive performance. We identified a novel mobile genetic element defined by a previously unrecognized integrase related to the tyrosine integrase/recombinase family. The genomic context of these integrases indicates that their activity contributes to generating genomic diversity among globally abundant marine picocyanobacteria. We identified representative sequences of this integrase in cultured isolate and single-cell genomes of *Prochlorococcus* and *Synechococcus* and found that the region immediately surrounding the integrase represents a genomic island whose length, gene content, and gene orientation varies among individual genomes. Variable genes found near the integrase include putative restriction/modification systems, biosynthetic enzymes, and nutrient acquisition genes, indicating that the integrase-associated element can move genetic cargo of ecological relevance in the ocean. The consistent proximity of the integrase to two specific tRNAs suggests these as likely integration sites for the element. The integrases are also frequently, though not exclusively, found near a small serine recombinase which might contribute to resolving mobile element insertion into a target molecule^46^. However, the specific mechanism through which this element is mobilized or integrated is not yet known.

Additionally, proteins in the integration and excision functional category are related to the viral capacity to integrate its genome into a host. These proteins are of particular interest in viral profiling studies as they are used to distinguish between lytic and temperate viral life-cycles^13,16,47^. We note that mobile genetic elements, some of which contain integrases, are widespread in environmental samples and are still being discovered and characterized^37,38^. Understanding the origins and dynamics of these elements remains an open question. Finally, the ability to detect proteins that function in host genome integration is crucial for the field of phage therapy^48^.

Aside from serving as feature embeddings for protein sequences in downstream tasks, PLM transformation of viral proteins also captured underlying patterns of protein functions in viruses. The clustering together of families that function in virion formation and host lysis separately from families involved in metabolic processes, expression regulation, and host genome integration constitutes a biologically meaningful division of viral function. While our original hypothesis was that sequences in different VPFs but the same functional category would help predict unseen sequences of that function, reflecting shared evolution or functional redundancy in reticulate evolution, we were surprised that using sequences in related functions also aids in prediction. Having identified that PLM embeddings capture this biology, we show that PLM-based representations outperform feature engineering and uninformed but local learning through convolution for PVP classification. As PVPs are crucial to initial phage-host interaction, the ability to better identify this class of proteins in UViGs will aid studies of phage-host specificity.

Our study must acknowledge several limitations. In attempting to systematically annotate VPF function and highlight the ability to label integration and excision VPFs, we note that for experimentalists interested in annotating UViGs there are a plethora of methods, parameters, and thresholds to decide, and they may arrive at an annotation for a specific gene not annotated in large-scale approaches by thorough investigation. Annotation goals are project-specific and may have different levels of granularity needed for annotation; here we have focused on protein family level annotations. In selecting the PHROGs database for training the functional classifier we benefited from the high-level functional category annotation as it collapses a wide array of annotation terms into defined categories. However, the categories vary in their scope and while some are relatively narrow (integration and excision; lysis) and their prediction can be relevant to experimentalists, the ones that are comparatively broad (head and packaging; moron, auxiliary metabolic gene and host takeover) are limited in their ability to provide specific information when predicted. Finally, while we leverage previously trained PLMs due to the computational resources necessary to train large language models and as proof of their ability to capture viral protein function, there may be more optimal approaches to train PLMs for viral function prediction. Future work will seek to determine whether there are supervised tasks that can increase performance of the functional classifier.

Our PLM-based classifier is trained on the same data that underlies the PHROG pHMMs yet can detect homology across a larger sequence space, identifying integrase genes that the original pHMMs and other annotation tools were blind to. This suggests that PLMs are accessing features of sequence space that alignment-based methods cannot. We hypothesize that these features reflect protein structure, as PLMs and large language models more generally, have been shown to be adept at capturing domain and structural features of proteins. Using our approach, targeted hypotheses about protein function can be gleamed from PLM-based classification and then tested experimentally, providing a powerful method for directing study into currently hidden functions of interest.

## Methods

### Viral protein sequence data

The PHROGs VPF database v3^21^ (https://phrogs.lmge.uca.fr/) was downloaded on 01/26/2022. Reannotation data was downloaded after v4 release. EFAM VPF database^22^ was downloaded from project repository on CyVerse Data Commons on 09/07/2022. PHANNs protein sequences and annotations^35^ (https://phanns.com/downloads) was downloaded on 01/17/2023.

### Protein language models

Protein sequences were embedded to vectors using trained PLMs. Transformer_BFD PLM from the ProtTrans^28^ project was used via the DeepChainBio/BioTransformers python package. Sequences were embedded with pool_mode=‘mean’ and batch_size=2. Sequences were cut off at 5,096 amino acids which is the limit of the Transformer_BFD PLM. LSTM_Uniref90 and LSTM_Uniref90_MT from the ProSE^30^ project were download from the project github repository (https://github.com/tbepler/prose) and protein sequences were embedded with the embed_sequences.py script with –pool avg. Transformer Uniref90 MT from the ProteinBERT^29^ project was downloaded from the project github repository (https://github.com/nadavbra/protein_bert) and protein sequences were embedded using the get_model_with_hidden_layers_as_outputs function in the proteinbert python package. All protein sequence embedding was performed on 2 NVIDIA TITAN V GPUs.

### Classifier training and evaluation

To test the ability of a model to predict functional category for a test sequence, all labeled PHROG families were split into five, stratified sets for fivefold cross-validation. In each split, training was done on all sequences in training families while testing was performed on a single randomly selected sequence from the testing families. Data preparation for model training was done using sklearn^49^ methods StratifiedKFold and LabelBinarizer. The same training-validation procedure was used for cluster1 vs. cluster2 fivefold cross-validation.

For training the PHROGs functional category classifier used in the EFAM classification experiment, families from the ‘unknown function’ category were included as an additional functional category. However, because the unknown families may be missing annotation, any family that was predicted by the model trained without the unknown function category with a score >0.5 was removed from training (n=19,512).

The classifier architecture is a dense, feed-forward neural network trained with tensorflow^50^. The network has three hidden layers of dimensions 512, 256, and 128 trained with 20% dropout and ReLU activation. The output layer is of dimension equal to the number of functional categories being predicted and has a softmax activation. Input dimension is equal to the embedding vector length output from the PLM. For PLMs with embedding dimension greater than 1,024, an additional hidden layer of dimension 1,024 was added as the first hidden layer. The model was fit with the following parameters: n_epoch=20, loss=categorical_crossentropy, opt=Adam(0.0001), batch size=60. Class prediction is assigned based on the highest probability of the softmax layer. For binary classifiers based on binary clusters of PHROGs functional categories, the same architecture and training parameters are used with the exception of n_epochs=5.

Evaluation for the classifier was measured per-functional category using area under the receiver operating characteristic curve (AUROC), area under precision-recall curve (AUPRC), and the F1-score: $F_{1}=2\cdot \frac{TP}{TP+\frac{1}{2}(FP+FN)}$, where TP, FP, and FN are the number of true positive, false positive, and false negatives predicted, respectively. ROC and PRC curves, AUC, and F1-score were all calculated using sklearn^49^ methods roc_curve, precision_recall_curve, and auc. In the case of PHROGs fivefold cross-validation, true labels are known for holdout families. In the case of EFAM, true labels are assigned based on HMM matching of EFAM families to PHROG families. EFAM families were aligned using clustal omega v1.2.4^51^ and searched against the PHROG HMM database using hhsearch^52^. PHROGs functional label assignment was made if an EFAM family matched a PHROGs HMM with e-value < 1.0*e*^−12^. The label of the PHROGs family with the lowest e-value is considered the true label unless that label is unknown function in which case the next lowest family label is assigned. For predicting EFAM category in the absence of PHROGs HMM hits, the decision threshold probability for category assignment in EFAM was identified by calculating the per-category maximum F1-score. For EFAM VPFs with annotation in the EFAM database, annotation terms present > 5 times in families predicted by the classifier as integration and excision are shown to highlight the split around proteins of length 100 in the category.

### Viral protein family embedding space

PHROGs v4 annotation were used for interrogation of the embedding space. PHROGs families were collapsed to centroid vectors by taking the column average of the vector representation of all proteins in a family. Uniform Manifold Approximation and Projection for Dimension Reduction (UMAP) in python^53^ was used to visualize embedded VPFs. Cosine similarity is a measure of similarity between two vectors and is calculated:

$$\text{cosine similarity }=\frac{\sum_{i=1}^{n}{\text{u}}_{i}{\text{v}}_{i}}{\sum_{i=1}^{n}{\text{u}}_{i}\sum_{i=1}^{n}{\text{v}}_{i}}$$

where **u** and **v** are vectors of length *n* and *x*_*i*_ is the *i*-th element of each vector. It is used to measure sequencesequence similarity and family-family similarity from protein vectors and family centroid vectors, respectively. Families with vector similarities > 0.999 (n=312) were excluded from median family mean sequence-sequence similarity calculation as some families have only duplicate sequences as PHROGs did not deduplicate protein sequences. For intra-category similarity, pairwise similarity was calculated for all category families. For inter-category similarity, each family in one category was compared to each family in another category with the mean across all pairwise comparisons constituting the category-category similarity. Differences in the distribution of similarities between categories were evaluated with the independent student t-test with Bonferroni correction using statannotations^54^. The category-category similarity matrix was converted to a network using networkx^55^ and displayed with spectral layout. The distance matrix was clustered using sklearn^49^ SpectralClustering with n_clusters=2.

### Phage virion protein classification

PHANNs protein sequences were embedded using the Transformer_BFD PLM and a PVP vs other classifier was trained with the same architecture and parameters as the cluster1 vs. cluster2 classifier. Training and testing sequence split is as described previously^34^. All sequences in the 10 PHANNs validation splits for all PVP classes are combined to a single PVP training set (n=154,183) and all 10 other validation splits were combined to a single other training set (n=336,151). Testing was done on the held PVP sequences for all classes (n=14,477) and the held out other sequences (n=33,402).

### Viral protein sequence annotation validation tools

Viral sequence predictions were manually validated using existing sequence and structural homology software. Individual sequence homology was performed with NCBI-hosted blastp^56^ using the nr database and default parameters. Domain prediction was performed using InterPro^57^. MPI bioinformatics suite^58^ was used for searching protein sequences against HMM databases using HHpred^59^ with default databases (PDB_mmCIF30_10_Jan, UniProt-SwissProt-viral70_3_Nov_2021, COG_KOG_v1.0, PHROGs_v4) and parameters and for searching sequence databases (nr30_17_jan) for HMM hits using HMMER^60^ with default parameters. Phyre2 was used for protein structural fold prediction and 3D model prediction^61^.

### Investigation of predicted integrase protein families

A putative integrase protein sequence (MAK08069.1) from cluster158946 was used to search MGniFY for similar sequences in metagenomic datasets. We took the first MGnify hit, MGYP000503484273 (e-value 3.3E-257), and used it as a seed to search the IMG-VR database for sequences in the Viral Protein Database using default cutoffs (1E-5). This led to the discovery of putative integrase homologs from *Prochlorococcus* and *Synechococcus* genomes, which were interrogated further.

### Integrase analysis

Novel integrase sequences originally identified in IMG were used to query a custom database of *Prochlorococcus* genomes from cultured isolates and single cell genomes^37^. Additional sequences, such as those from *Synechococcus*, were retrieved through blastp searches of the NCBI nr database (Supplemental Data 3). The tyrosine integrase phylogeny was constructed from a set of tyrosine recombinases extracted from the UniRef50 database (http://www.uniprot.org/uniref) using HMM models from ref^39^; a set of integrases associated with *Prochlorococcus* Tycheposons and cryptic elements^37^; and representative sequences VEIME-associated integrases^38^ (based on 40% identity clusters generated with MMSeqs2^62^). Sequences were aligned with Mafft v7.520^63^, a maximum likelihood phylogeny was generated using FastTree v2.1.11^64^, and the tree was plotted using iTOL^65^. Genome regions surrounding the integrases were plotted in R using gggenomes 0.9.7.9000 (https://github.com/thackl/gggenomes).

### Protein structure modeling of a novel integrase sequence

Protein structure can be conserved among very distantly related sequences. We previously utilized homology modeling approaches to identify distantly related structural homologs to novel viral capsid protein sequences^66^. Here, we took a similar approach to identify structures related to sequences in our putative novel integrase family. We utilized the fully automated protein structure homology-modelling server SWISS-MODEL via the Expasy web server^67^ for template selection, target/template alignment, and model generation using default parameters for an integrase sequence from the Prochlorococcus PAC1 genome (WP 052038630). The top template, as identified by the Global Model Quality Estimate score, was PDB ID 1Z1B, the phage lambda integrase^68^. The target/template alignment has 13% sequence identity, consistent with our sequences not previously being identified as integrases. The MolProbity protein quality score, provided by SWISS-MODEL, which combines protein structure quality features that together reflect crystallographic resolution, was 2.2^69^. The lambda integrase is a tyrosine recombinase with defined active site residues Arg 212, Lys 235, His 308, Arg 311, His 333, and Tyr 342^68^. In a study of catalysis requirements for tyrosine recombinases, the key residues strictly required for function were identified as the Tyr (Y) and Lys (K) residues^40^. The target/template alignment demonstrates that residues Arg 212, Lys 235, Arg 311, and Tyr 342 are conserved in our target sequence (Supplemental Figure 4, panel A). The sequence is modeled as a homo-tetramer, consistent with the quaternary structure of the template (Supplemental Figure 4, panel B).

## Figures and Tables

**Table 1:** PLM-based viral protein sequence embedding produces best performance in phage virion protein (PVP) classification task. PVP classification task designed previously^34^ with PHANNs dataset^35^.

Method	Recall (%)	Precision (%)	F1-score (%)
PLM+FNN	90.32	96.88	93.48
*DeePVP	88.10	96.75	92.22
*PHANNs	91.68	76.11	83.17

* is performance reported previously^34^.

## Data Availability

Protein sequence embeddings for PHROGs, EFAM, and PhaNNs are available for download from google cloud. See README on the project repository https://github.com/kellylab/viral-protein-function-annotation-with-proteinlanguage-model for details on data download.

## References

[1] Roux S. Ecogenomics and potential biogeochemical impacts of globally abundant ocean viruses. Nature, 537(7622):689–693, 2016. 27654921 10.1038/nature19366

[2] Paez-Espino D. Uncovering Earth’s virome. Nature, 536(7617):425–430, 2016. 27533034 10.1038/nature19094

[3] Gregory A. C. Marine DNA Viral Macro- and Microdiversity from Pole to Pole. Cell, 177(5):1109–1123.e14, may 2019. 31031001 10.1016/j.cell.2019.03.040 PMC6525058

[4] ter Horst A. M. Minnesota peat viromes reveal terrestrial and aquatic niche partitioning for local and global viral populations. Microbiome, 9(1):233, 2021. 34836550 10.1186/s40168-021-01156-0 PMC8626947

[5] Gregory A. C. The gut virome database reveals age-dependent patterns of virome diversity in the human gut. Cell Host & Microbe, 28(5):724–740.e8, 2020. 32841606 10.1016/j.chom.2020.08.003 PMC7443397

[6] Camarillo-Guerrero L. F. Massive expansion of human gut bacteriophage diversity. Cell, 184(4):1098–1109.e9, 2021. 33606979 10.1016/j.cell.2021.01.029 PMC7895897

[7] Nayfach S. Metagenomic compendium of 189,680 DNA viruses from the human gut microbiome. Nature Microbiology, 6(7):960–970, 2021. 10.1038/s41564-021-00928-6 PMC8241571 34168315

[8] Roux S. Virsorter: mining viral signal from microbial genomic data. PeerJ, 3:e985, May 2015. 26038737 10.7717/peerj.985 PMC4451026

[9] Ren J. VirFinder: a novel k-mer based tool for identifying viral sequences from assembled metagenomic data. Microbiome, 5(1):69, 2017. 28683828 10.1186/s40168-017-0283-5 PMC5501583

[10] Ren J. Identifying viruses from metagenomic data using deep learning. Quantitative Biology, 8(1):64–77, 2020. 34084563 10.1007/s40484-019-0187-4 PMC8172088

[11] Wood D. E. Improved metagenomic analysis with Kraken 2. Genome Biology, 20(1):257, 2019. 31779668 10.1186/s13059-019-1891-0 PMC6883579

[12] Guo J. VirSorter2: a multi-classifier, expert-guided approach to detect diverse DNA and RNA viruses. Microbiome, 9(1):37, 2021. 33522966 10.1186/s40168-020-00990-y PMC7852108

[13] Kieft K. VIBRANT: automated recovery, annotation and curation of microbial viruses, and evaluation of viral community function from genomic sequences. Microbiome, 8(1):90, 2020. 32522236 10.1186/s40168-020-00867-0 PMC7288430

[14] Tisza M. J. Cenote-Taker 2 democratizes virus discovery and sequence annotation. Virus Evolution, 7(1):veaa100, jan 2021. 33505708 10.1093/ve/veaa100 PMC7816666

[15] Glickman C. Simulation study and comparative evaluation of viral contiguous sequence identification tools. BMC Bioinformatics, 22(1):329, 2021. 34130621 10.1186/s12859-021-04242-0 PMC8207588

[16] Camargo A. P. You can move, but you can’t hide: identification of mobile genetic elements with genomad. bioRxiv, 2023.

[17] Meier-Kolthoff J. P. and Göker M.. VICTOR: genome-based phylogeny and classification of prokaryotic viruses. Bioinformatics, 33(21):3396–3404, 07 2017. 29036289 10.1093/bioinformatics/btx440 PMC5860169

[18] Bin Jang H. Taxonomic assignment of uncultivated prokaryotic virus genomes is enabled by gene-sharing networks. Nature Biotechnology, 37(6):632–639, 2019. 10.1038/s41587-019-0100-8 31061483

[19] Moraru C.. Virclust – a tool for hierarchical clustering, core gene detection and annotation of (prokaryotic) viruses. bioRxiv, 2021. 10.3390/v15041007 PMC10143988 37112988

[20] Pons J. C. VPF-Class: taxonomic assignment and host prediction of uncultivated viruses based on viral protein families. Bioinformatics, 37(13):1805–1813, 01 2021. 33471063 10.1093/bioinformatics/btab026 PMC8830756

[21] Terzian P. PHROG: families of prokaryotic virus proteins clustered using remote homology. NAR Genomics and Bioinformatics, 3(3), 08 2021. lqab067. 10.1093/nargab/lqab067 PMC8341000 34377978

[22] Zayed A. A. efam: an expanded, metaproteome-supported HMM profile database of viral protein families. Bioinformatics, 37(22):4202–4208, 06 2021. 34132786 10.1093/bioinformatics/btab451 PMC9502166

[23] Abdelkareem A. O. Virnet: Deep attention model for viral reads identification. In 2018 13th International Conference on Computer Engineering and Systems (ICCES), pp. 623–626, 2018.

[24] Tynecki P. Phageai - bacteriophage life cycle recognition with machine learning and natural language processing. bioRxiv, 2020.

[25] Asgari E. and Mofrad M. R. K.. Continuous distributed representation of biological sequences for deep proteomics and genomics. PLOS ONE, 10(11):1–15, 11 2015. 10.1371/journal.pone.0141287 PMC4640716 26555596

[26] Heinzinger M. Modeling aspects of the language of life through transfer-learning protein sequences. BMC Bioinformatics, 20(1):723, 2019. 31847804 10.1186/s12859-019-3220-8 PMC6918593

[27] Rives A. Biological structure and function emerge from scaling unsupervised learning to 250 million protein sequences. Proceedings of the National Academy of Sciences, 118(15):e2016239118, 2021. 10.1073/pnas.2016239118 PMC8053943 33876751

[28] Elnaggar A. Prottrans: Towards cracking the language of lifes code through self-supervised deep learning and high performance computing. IEEE Transactions on Pattern Analysis and Machine Intelligence, pp. 1–1, 2021.31331880

[29] Brandes N. ProteinBERT: a universal deep-learning model of protein sequence and function. Bioinformatics, 01 2022. btac020. 10.1093/bioinformatics/btac020 PMC9386727 35020807

[30] Bepler T. and Berger B.. Learning the protein language: Evolution, structure, and function. Cell Systems, 12(6):654–669.e3, 2021. 34139171 10.1016/j.cels.2021.05.017 PMC8238390

[31] Nasir A. and Caetano-Anollés G.. A phylogenomic data-driven exploration of viral origins and evolution. Science Advances, 1(8):e1500527, 2015. 26601271 10.1126/sciadv.1500527 PMC4643759

[32] Balaji S. and Srinivasan N.. Comparison of sequence-based and structure-based phylogenetic trees of homologous proteins: Inferences on protein evolution. Journal of Biosciences, 32(1):83–96, 2007. 17426382 10.1007/s12038-007-0008-1

[33] Meng C. Review and comparative analysis of machine learning-based phage virion protein identification methods. Biochimica et Biophysica Acta (BBA) - Proteins and Proteomics, 1868(6):140406, 2020. 32135196 10.1016/j.bbapap.2020.140406

[34] Fang Z. DeePVP: Identification and classification of phage virion proteins using deep learning. Giga-Science, 11, 08 2022. giac076. 10.1093/gigascience/giac076 PMC9366990 35950840

[35] Cantu V. A. Phanns, a fast and accurate tool and web server to classify phage structural proteins. PLOS Computational Biology, 16(11):1–18, 11 2020. 10.1371/journal.pcbi.1007845 PMC7660903 33137102

[36] Mizuno C. M. Genomes of abundant and widespread viruses from the deep ocean. mBio, 7(4):e00805–16, 2016. 27460793 10.1128/mBio.00805-16 PMC4981710

[37] Hackl T. Novel integrative elements and genomic plasticity in ocean ecosystems. Cell, 186(1):47–62.e16, 2023. 36608657 10.1016/j.cell.2022.12.006

[38] Eppley J. M. Marine viral particles reveal an expansive repertoire of phage-parasitizing mobile elements. Proceedings of the National Academy of Sciences, 119(43):e2212722119, 2022. 10.1073/pnas.2212722119 PMC9618062 36256808

[39] Smyshlyaev G. Sequence analysis of tyrosine recombinases allows annotation of mobile genetic elements in prokaryotic genomes. Molecular Systems Biology, 17(5):e9880, 2021. 34018328 10.15252/msb.20209880 PMC8138268

[40] Gibb B. Requirements for catalysis in the Cre recombinase active site. Nucleic Acids Research, 38(17):5817–5832, 05 2010. 20462863 10.1093/nar/gkq384 PMC2943603

[41] Williams K. P.. Integration sites for genetic elements in prokaryotic tRNA and tmRNA genes: sublocation preference of integrase subfamilies. Nucleic Acids Research, 30(4):866–875, 02 2002. 11842097 10.1093/nar/30.4.866 PMC100330

[42] Koonin E. V. The global virome: How much diversity and how many independent origins? Environmental Microbiology, 25(1):40–44, 2023. 36097140 10.1111/1462-2920.16207

[43] Shen A. and Millard A.. Phage genome annotation: Where to begin and end. PHAGE, 2(4):183–193, 2021. 36159890 10.1089/phage.2021.0015 PMC9041514

[44] Borodovich T. Phage-mediated horizontal gene transfer and its implications for the human gut microbiome. Gastroenterology Report, 10, 04 2022. goac012. 10.1093/gastro/goac012 PMC9006064 35425613

[45] Jumper J. Highly accurate protein structure prediction with alphafold. Nature, 596:583–589, 8 2021. 34265844 10.1038/s41586-021-03819-2 PMC8371605

[46] Nicolas E. The tn¡i¿3¡/i¿-family of replicative transposons. Microbiology Spectrum, 3(4):3.4.14, 2015. 10.1128/microbiolspec.MDNA3-0060-2014 26350313

[47] Mavrich T. N. and Hatfull G. F.. Bacteriophage evolution differs by host, lifestyle and genome. Nature Microbiology, 2, 7 2017. 10.1038/nmicrobiol.2017.112 PMC5540316 28692019

[48] Pires D. P. Current challenges and future opportunities of phage therapy. FEMS Microbiology Reviews, 44(6):684–700, 05 2020. 32472938 10.1093/femsre/fuaa017

[49] Pedregosa F. Scikit-learn: Machine learning in Python. Journal of Machine Learning Research, 12:2825–2830, 2011.

[50] Abadi M. TensorFlow: Large-scale machine learning on heterogeneous systems, 2015.

[51] Sievers F. and Higgins D. G.. Clustal omega for making accurate alignments of many protein sequences. Protein Science, 27(1):135–145, 2018. 28884485 10.1002/pro.3290 PMC5734385

[52] Steinegger M. HH-suite3 for fast remote homology detection and deep protein annotation. BMC Bioinformatics, 20(1):473, 2019. 31521110 10.1186/s12859-019-3019-7 PMC6744700

[53] McInnes L. Umap: Uniform manifold approximation and projection for dimension reduction, 2018.

[54] Charlier F. Statannotations, October 2022.

[55] Hagberg A. A. Exploring network structure, dynamics, and function using networkx. In Varoquaux G. , editors, Proceedings of the 7th Python in Science Conference, pp. 11 – 15, Pasadena, CA USA, 2008.

[56] Altschul S. F. Gapped BLAST and PSI-BLAST: a new generation of protein database search programs. Nucleic Acids Research, 25(17):3389–3402, 09 1997. 9254694 10.1093/nar/25.17.3389 PMC146917

[57] Paysan-Lafosse T. InterPro in 2022. Nucleic Acids Research, 51(D1):D418–D427, 11 2022. 10.1093/nar/gkac993 PMC9825450 36350672

[58] Gabler F. Protein sequence analysis using the mpi bioinformatics toolkit. Current Protocols in Bioinformatics, 72(1):e108, 2020. 33315308 10.1002/cpbi.108

[59] Zimmermann L. A completely reimplemented mpi bioinformatics toolkit with a new hhpred server at its core. Journal of Molecular Biology, 430(15):2237–2243, 2018. Computation Resources for Molecular Biology. 29258817 10.1016/j.jmb.2017.12.007

[60] Potter S. C. HMMER web server: 2018 update. Nucleic Acids Research, 46(W1):W200–W204, 06 2018. 29905871 10.1093/nar/gky448 PMC6030962

[61] Kelley L. A. The phyre2 web portal for protein modeling, prediction and analysis. Nature Protocols, 10:845–858, 6 2015. 25950237 10.1038/nprot.2015.053 PMC5298202

[62] Steinegger M. and Soding J.. Mmseqs2 enables sensitive protein sequence searching for the analysis of massive data sets. Nature Biotechnology, 35:1026–1028, 11 2017. 10.1038/nbt.3988 29035372

[63] Katoh K. and Standley D. M.. MAFFT Multiple Sequence Alignment Software Version 7: Improvements in Performance and Usability. Molecular Biology and Evolution, 30(4):772–780, 01 2013. 23329690 10.1093/molbev/mst010 PMC3603318

[64] Price M. N. Fasttree 2 – approximately maximum-likelihood trees for large alignments. PLOS ONE, 5(3):1–10, 03 2010. 10.1371/journal.pone.0009490 PMC2835736 20224823

[65] Letunic I. and Bork P.. Interactive Tree Of Life (iTOL) v5: an online tool for phylogenetic tree display and annotation. Nucleic Acids Research, 49(W1):W293–W296, 04 2021. 33885785 10.1093/nar/gkab301 PMC8265157

[66] Kauffman K. M. Viruses of the Nahant Collection, characterization of 251 marine Vibrionaceae viruses. Scientific Data, 5(1):180114, 2018. 29969110 10.1038/sdata.2018.114 PMC6029569

[67] Waterhouse A. SWISS-MODEL: homology modelling of protein structures and complexes. Nucleic Acids Research, 46(W1):W296–W303, 05 2018. 29788355 10.1093/nar/gky427 PMC6030848

[68] Biswas T. A structural basis for allosteric control of dna recombination by integrase. Nature, 435:1059–1066, 6 2005. 15973401 10.1038/nature03657 PMC1809751

[69] Chen V. B. *MolProbity*: all-atom structure validation for macromolecular crystallography. Acta Crystallographica Section D, 66(1):12–21, Jan 2010. 10.1107/S0907444909042073 PMC2803126 20057044

